# Identifying hub functions and collaboration patterns in hikikomori support networks: A nationwide cross‐sectional study

**DOI:** 10.1002/pcn5.70311

**Published:** 2026-03-01

**Authors:** Masahide Usami, Yuki Mizumoto, Kumi Inazaki, Yuki Hakoshima, Kotoe Itagaki, Keita Yamamoto, Tetsushi Tsujimoto, Masao Yamasaki, Kazuhiko Saito

**Affiliations:** ^1^ Department of Child and Adolescent Psychiatry, National Kohnodai Medical Center Japan Institute for Health Security Ichikawa Japan; ^2^ Department of Clinical Psychology, National Kohnodai Medical Center Japan Institute for Health Security Ichikawa Japan; ^3^ Department of Social Work, National Kohnodai Medical Center Japan Institute for Health Security Ichikawa Japan; ^4^ Shiga Prefectural Mental Health and Welfare Center Shiga Japan; ^5^ Kochi Prefectural Mental Health and Welfare Center Kochi Japan; ^6^ Aiiku Research Institute Imperial Gift Foundation Boshi‐Aiiku‐Kai Tokyo Japan

**Keywords:** hikikomori, interorganizational collaboration, public health systems, service accessibility, social withdrawal

## Abstract

**Aim:**

Hikikomori, a condition involving prolonged social withdrawal, has become a major public health and social challenge in Japan and many countries. While support institutions are essential for reaching this population, little is known about how their organizational characteristics influence collaboration with other services.

**Methods:**

We conducted a nationwide cross‐sectional survey of 1024 hikikomori‐related support institutions in Japan between February and March 2021. Institutions were classified using K‐means cluster analysis based on staff size, collaboration count, and case volume. Multiple regression models assessed associations between these attributes and levels of external collaboration with health and welfare organizations.

**Results:**

Three distinct institutional clusters were identified. Institutions serving more diverse populations showed higher levels of interorganizational collaboration; a lean, outreach‐oriented “Collaborative Core” subgroup exhibited the strongest external engagement.

**Conclusion:**

Structural features such as diversity, functional capacity, and outreach orientation were associated with network collaboration. Policymakers and administrators should consider these structural attributes when designing inclusive and coordinated public mental health services for socially withdrawn populations. The proposed framework may serve as a model for similar contexts across countries.

## BACKGROUND

In fiscal year (Japan) (FY)2022, the Ministry of Education recorded over 299,000 elementary and junior high school students in Japan as school nonattendance.^1^ Hikikomori intersects with broader social issues, including aging parents caring for socially withdrawn adult children.^2^, ^3^, ^4^ Although hikikomori is not currently classified as a formal diagnosis in International Classification of Diseases, 11th Revision (ICD‐11), it overlaps with categories related to social withdrawal, raising classification and intervention challenges.^5^, ^6^ Hikikomori, a term originating in Japan, refers to a prolonged state of severe social withdrawal, in which individuals remain in their homes and avoid social participation for at least 6 months. Initially conceptualized as a culture‐bound syndrome affecting adolescents and young adults in Japan during the 1990s, hikikomori has since been recognized as a multifaceted psychosocial condition with global relevance.^7^, ^8^, ^9^, ^10^ It transcends age, sex, and cultural boundaries. It is increasingly seen not merely as a symptom but as a syndrome, characterized by sustained social isolation, functional impairment, and comorbid mental health issues such as depression, anxiety, and avoidant personality traits.^11^

In Japan, this phenomenon has evolved beyond its youth‐centric origins to become a pressing social issue affecting middle‐aged and older adults. The so‐called “8050 problem”—a term referring to parents in their 80s still supporting socially withdrawn children in their 50s—has attracted public attention and reflects the intergenerational nature and chronicity of the problem. According to the 2023 Cabinet Office survey, approximately 1.46 million individuals aged 15–64 years are estimated to be living in hikikomori conditions in Japan, highlighting its scale and persistence. In parallel, the Ministry of Education reported record‐high levels of school nonattendance in FY2022.^12^ These trends suggest that the growing population is at risk of social isolation and functional decline across multiple life stages. Globally, hikikomori is gaining recognition as an emerging mental health and social challenge. A previous study argued that hikikomori should no longer be considered a culture‐bound syndrome but a globally relevant, transdiagnostic condition. Research from South Korea, Hong Kong, Italy, Spain, and the United States has documented similar patterns of extreme withdrawal, although prevalence rates, diagnostic frameworks, and service structures vary widely.^11^ A bibliometric analysis revealed a substantial increase in hikikomori‐related publications over the past two decades, with an emerging shift toward intervention‐focused and comparative cross‐cultural studies. A previous study also emphasized the need to harmonize definitions and develop a consensus on diagnostic criteria to facilitate international research collaboration.

Despite the growing international attention, hikikomori remains difficult to define and diagnose.^11^, ^13^, ^14^ Its boundaries with other psychiatric conditions, such as schizophrenia spectrum disorder, autism spectrum disorder (ASD), and major depressive disorder, are often blurred. Some individuals meet the full criteria for a psychiatric diagnosis, whereas others do not.^15^, ^16^, ^17^, ^18^, ^19^ The latest Japanese guidelines define hikikomori behaviorally rather than diagnostically, including individuals with or without psychiatric comorbidities. This definitional flexibility allows inclusivity and introduces challenges in standardizing support frameworks and evaluating outcomes across systems.^20^ Beyond the clinical complexities, structural and technological changes have further complicated the hikikomori setting. The coronavirus disease 2019 (COVID‐19) pandemic, as a global health emergency, has also accelerated social withdrawal by increasing home confinement and digital communication.^21^, ^22^, ^23^, ^24^, ^25^, ^26^, ^27^ Sales‐Filho et al.^28^ reviewed the relationship between hikikomori and digital media use and found that excessive reliance on online interactions may exacerbate social withdrawal among at‐risk populations, particularly adolescents. In Japan, where Internet access is ubiquitous and societal pressure toward conformity remains strong, such dynamics may further entrench isolation.^28^ In response to these growing concerns, the Japanese Ministry of Health, Labour and Welfare (MHLW) introduced the National Hikikomori Support Guidelines in 2010 and revised them in 2022. These policies emphasize early detection, multi‐sectoral collaboration, and family involvement. The supplementary handbook, released in 2025, provides practical tools for local governments and community agencies to implement coordinated outreach and support. However, there is substantial variability in how these policies are operationalized.^29^ Multiple entry points—mental health centers, educational institutions, social welfare offices, and nonprofit organizations (NPOs)—mean responsibility is diffused across sectors, often without robust referral systems or shared protocols.^30^ Notably, the challenge of supporting hikikomori is clinical and structural in nature. Adequate support requires collaboration among diverse sectors, including psychiatry, education, public health, child welfare, and employment services.^28^ However, institutional silos, mismatched eligibility criteria, and divergent professional paradigms hinder seamless cooperation.^31^ In Japan, where administrative responsibilities are stratified by the ministry and municipality, coordination among sectors is often ad hoc and personnel‐dependent. Even well‐resourced facilities may have limited resources for complementary services because of jurisdictional boundaries or a lack of formal agreements. Empirical evidence on the function of hikikomori support systems remains limited.^32^ Although case reports and qualitative studies have shed light on family dynamics and psychosocial trajectories, little is known about the institutional characteristics that promote effective collaboration. Neoh et al. (2023) proposed more research on system‐level practices and organizational networks to complement individual‐focused interventions.^8^ Understanding how different types of facilities—public health centers, schools, and non‐governmental organizations (NGOs), among others—coordinate their activities and what structural factors predict strong collaboration is essential for designing scalable models of care.^32^, ^33^, ^34^ To fill this gap, this study draws on a large‐scale institutional survey conducted in Japan in 2021 as part of the MHLW's Social Welfare Promotion Program. The project was initially scheduled to begin in 2020, but was delayed owing to the COVID‐19 pandemic. All meetings were held online, and data were collected using Microsoft Forms between February and March 2021. The survey targeted many institutions involved in hikikomori‐related work, including Hikikomori Regional Support Centers, Public Health Centers, Educational Support Centers, Child Guidance Centers, and NPOs. A total of 1024 institutional responses were collected, making it one of the most comprehensive datasets on hikikomori support structures. This study analyzed an institutional dataset to explore the structural factors associated with interorganizational collaboration. We focused on four key predictors: (1) institution type, (2) staff size, (3) diversity of the supported population, and (4) number of hikikomori cases handled. We hypothesize that institutions fulfilling “hub” functions—specifically those serving diverse target populations and managing high case volumes—will exhibit stronger external collaboration networks, regardless of their staff size. Additionally, we conducted cluster analysis to identify the typologies of operational styles among the facilities. We examine these structural variables quantitatively to contribute to the evidence base for developing integrated community‐based hikikomori support systems within and beyond Japan.

### Rationale and hypotheses

We focused on structural predictors related to institutional capacity and function: institution type, staff size, client diversity, and case volume. We posited that diverse client needs necessitate multi‐sector engagement and that higher case volumes typically require broader referral networks. Accordingly, we hypothesized that (1) institutions serving more diverse clients report greater collaboration and (2) institutions handling larger case volumes show greater collaboration.

## METHODS

### Study design and data source

This study is a secondary analysis of data collected through a national survey titled “Questionnaire Survey for Hikikomori Support Providers (Institutional Edition),” conducted as part of the MHLW's FY2020 Social Welfare Promotion Project. The National Center for Global Health and Medicine led and implemented the project during the COVID‐19 pandemic, significantly delaying the original research schedule. All planning meetings were conducted online, and the survey was administered using Microsoft Forms. The targeted institutions included prefectural and municipal governments, Public Health Centers, Hikikomori Regional Support Centers, Child Guidance Centers, Educational Support Centers, Self‐Reliance Support Centers, NPOs related to hikikomori and school nonattendance (based on the Cabinet Office database), and Boards of Education. Given that some facilities serve multiple functions, category‐specific totals can exceed the overall *N* = 898.

The online questionnaire was opened from February 6 to March 9, 2021, following pre‐testing and stakeholder discussions. Data collection occurred during the COVID‐19 pandemic, which may have influenced institutional operations and collaboration patterns. We distributed the survey to approximately 9000 institutions nationwide, covering all municipalities, public health centers, welfare offices, and educational support centers involved in hikikomori support. We received valid responses from 1024 facilities, yielding a response rate of approximately 11.4%. Although we could not perform a detailed non‐response bias analysis due to the anonymity of the survey, the responding facilities were distributed nationwide, covering all 47 prefectures, which suggests a reasonable degree of representativeness.

### Sample selection and exclusions

We obtained 1024 valid institutional responses. We excluded facilities with missing values on the four key variables (collaboration count, total staff, total cases, and target diversity), yielding 988 facilities. We then limited the sample to facilities under the MHLW 12‐category classification (*N* = 902). Finally, we removed outliers on total staff (>150), resulting in a final analytic sample of 898 facilities. The outlier threshold was a priori based on the empirical distribution (Q3 = 6, 95th = 16, 99th = 45).

### Variables and measures

The questionnaire included items on institutional characteristics, human resources, service target populations, and interorganizational collaboration.

We extracted and defined the following variables:

Collaboration Count (Collab): The number of organizations each facility reported collaborating with based on 16 predefined categories (e.g., municipal departments, medical institutions, educational sectors, and NPOs). Each collaboration was coded as binary (1 = yes, 0 = no), and the sum was used as the total collaboration score. Staff Size (Staff): The total number of full‐time and part‐time personnel was calculated by summing responses from two sets of questionnaire items: general personnel (“main contact persons”) and professionals by role (psychologists, nurses, social workers, and physicians). Target Population Diversity Score: Seven target groups were listed in the questionnaire, including individuals with mental illness, suspected developmental disorders, victims of domestic violence, and older adults. We coded each as binary and summed them to produce a diversity score (range: 0–7). Case Volume (Cases): The total number of hikikomori cases managed by each facility in FY2019 (e.g., new and ongoing cases) as reported in the questionnaire.

### Facility type as a multi‐select attribute

Facility type is a multi‐select attribute; denominators equal the number of facilities that selected that type. Therefore, type‐stratified totals exceed the number of unique facilities (*N* = 898). Facilities self‐identified their organizational type(s) from 16 predefined categories, and multiple selections were allowed. Accordingly, analyses “by type” (e.g., Figure 4) use a function‐level dataset in which each facility‐type pair constitutes one observation; denominators are the number of facilities that selected that type. In contrast, clustering and all regressions were performed at the unique‐facility level (*N* = 898). Therefore, type‐stratified totals do not equal the number of unique facilities.

### Statistical analysis

Facilities with missing values on the four key variables (collaboration count, total staff, total cases, and target diversity) were excluded via listwise deletion. Missingness was low and largely item‐specific; listwise deletion was pre‐specified. Multiple‐imputation sensitivity checks yielded consistent inferences (Table S2). We also excluded outliers in total staff (>150) under a pre‐specified rule informed by distributional percentiles (Q3 = 6, 95th = 16, 99th = 45). Results were robust in a sensitivity analysis that retained these outliers (*N* = 902; Table S2).

Descriptive Statistics: Each key variable's distribution, means, and standard deviations.

Bivariate Analysis: Correlation matrices and scatterplots to examine relationships between variables.

### Model intent and scope

Analyses were conducted in R statistical software (R) (version 4.3.1, R Foundation for Statistical Computing). We specified a parsimonious, a priori structural model focusing on institutional features (staffing flexibility, client diversity, and case volume) as explanatory variables for collaboration. As pre‐specified, our goal was to identify structural correlates rather than to maximize predictive accuracy; the modest explained variance (*R* ^2^ = 0.039) is expected for facility‐level structural models.

We used multiple linear regression with heteroskedasticity‐robust (HC3) standard errors. As a sensitivity analysis, a Poisson/negative binomial specification produced substantively similar signs and significance (Table S2).

### Clustering analysis

Before clustering, all variables were standardized using the *Z*‐score transformation. The optimal number of clusters was determined by examining the elbow method and the silhouette scores, both of which supported a three‐cluster solution. Clustering used the final analytic sample (*N* = 898) after the exclusions described above. The average silhouette width at *K* = 3 was 0.41 (higher than *K* = 2 or 4), and the elbow curve showed a marked inflection at *K* = 3.

### Ethical considerations

This study was an institutional (organization‐level) survey of service providers and did not involve recruitment of hikikomori individuals, collection of patient/client data, or any intervention beyond routine professional activities. Therefore, it falls outside the scope of research requiring formal ethics committee review under applicable Japanese regulations. The survey was conducted as part of routine quality improvement and staff training/development activities in psychiatric services. All responses were collected in a non‐identifiable format and analyzed at the institutional level. Prior to survey access, respondents were informed that their responses would be anonymized and might be used for research dissemination and publication; submission of the completed survey indicated voluntary agreement (implied consent).

## RESULTS

Of the 1024 valid responses, 988 facilities had complete data on the four variables. Among these, 902 belonged to the MHLW 12 categories. After excluding four outlier facilities with a total staff count > 150, the final analytic sample comprised 898 facilities.

### Collaboration patterns by institution type

The cross‐tabulation of institution types and their reported collaboration partners revealed significant differences in interorganizational engagement. Core public institutions such as Mental Health and Welfare Centers, Public Health Centers, and Hikikomori Regional Support Centers exhibited the highest number of collaborations, with an average of more than 10 partners per facility. These institutions frequently collaborate across the health, welfare, education, and labor sectors. In contrast, institutions in the education sector, including Educational Support Centers and school‐affiliated entities, tend to have more limited collaboration networks, with lower engagement in cross‐sector partnerships. A heatmap visualization of collaboration frequencies across 16 partner types by institution category further illustrates these structural tendencies (Figure S2).

### Relationship between staff composition and collaboration

Multiple linear regression (unique‐facility level, *N* = 898) using standardized predictors showed that higher case volume (*β* = 0.149, *p* < 0.001) and greater target diversity (*β* = 0.116, *p* = 0.001) were associated with more external collaborations. In contrast, total staff was not significant (*β* = −0.023, *p* = 0.52). Model *R* ^2^ = 0.039.

### Hikikomori cases: Diversity and collaboration

A linear regression model demonstrated a positive association between the diversity of the target population and collaboration count. Specifically, each additional target category was associated with an average increase of 1.93 in collaboration count (*p* < 0.001).

### Case volume and collaboration

Facilities that managed higher case volumes in FY2019 also reported higher collaboration rates. The regression analysis revealed that for every additional case, the collaboration count increased by 0.09 on average (*p* < 0.001).

Furthermore, the total staff count (combined full‐ and part‐time) was strongly correlated with case volume (*r* = 0.98), indicating that staffing resources are closely tied to service capacity. However, because full‐ and part‐time staff counts were highly collinear (*r* = 0.86, variance inflation factor [VIF] = 4.05), it was challenging to disentangle their independent effects on outcomes beyond collaboration statistically.

### Cluster analysis: Typologies of facility operations

K‐means clustering using standardized staff scores, collaboration counts, and case volumes resulted in a three‐cluster solution that revealed distinct operational styles.

- Cluster 0: *N* = 252
- Staff mean: 3.5, collaboration mean: 4.7, and case mean: 9.3
- Cluster 1: *N* = 301
- Staff mean: 2.9, collaboration mean: 6.63, and case mean: 7.0
- Cluster 2: *N* = 345
- Staff mean: 11.0, collaboration mean: 7.6, and case mean: 53.7

This figure presents the interorganizational network based on reported collaborations among the 16 predefined facility types. Node size reflects the frequency of co‐occurrence (how often each facility type is selected as a partner). The edge width indicates the strength of the co‐occurrence (the number of facilities reported collaborating with both facility types). The proximity between nodes represents the closeness of their collaborative relationships as determined by the force‐directed layout algorithm.

**Figure 1:**
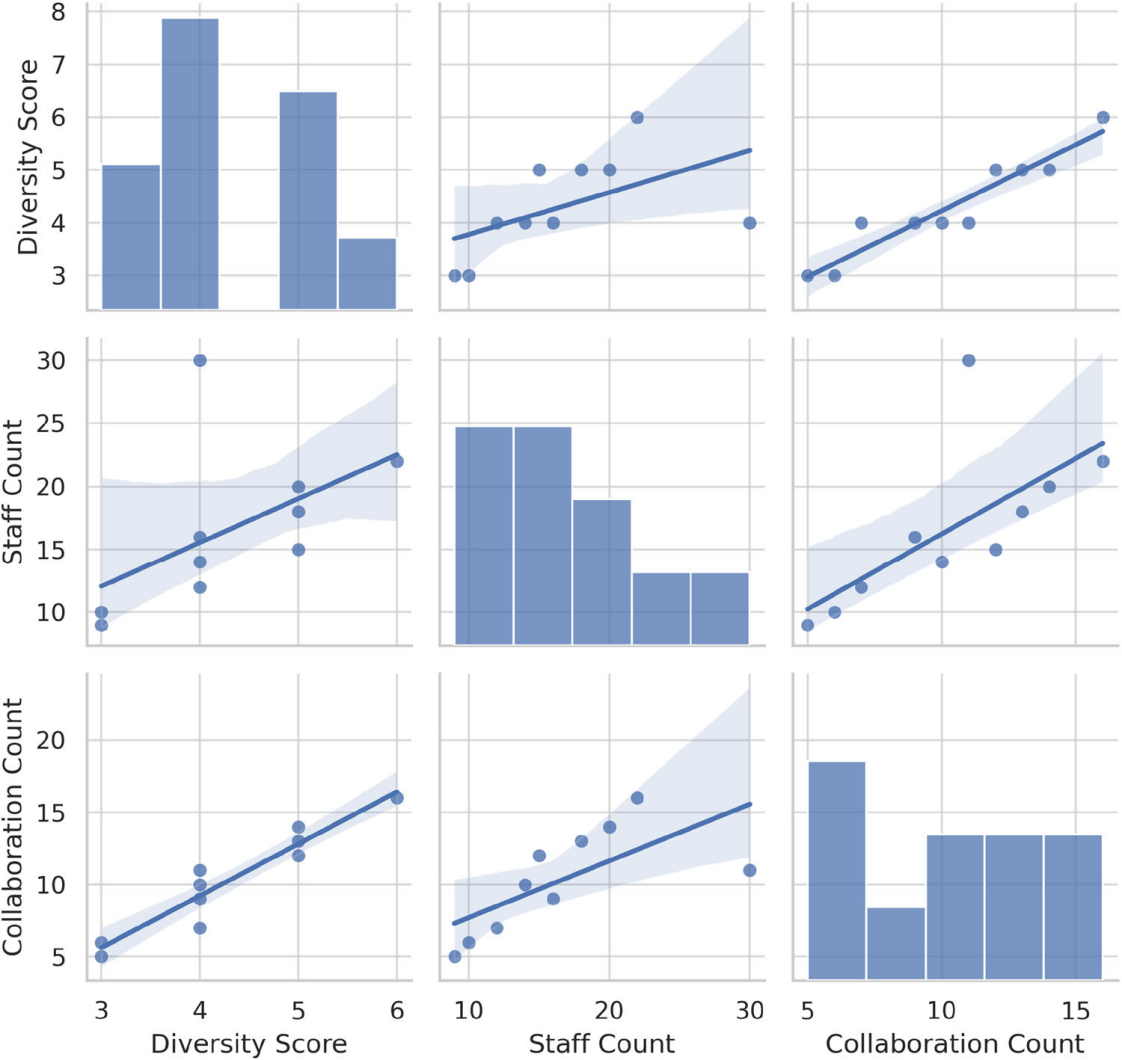
Scatterplot matrix of diversity score, staff count, and collaboration count.

**Figure 2:**
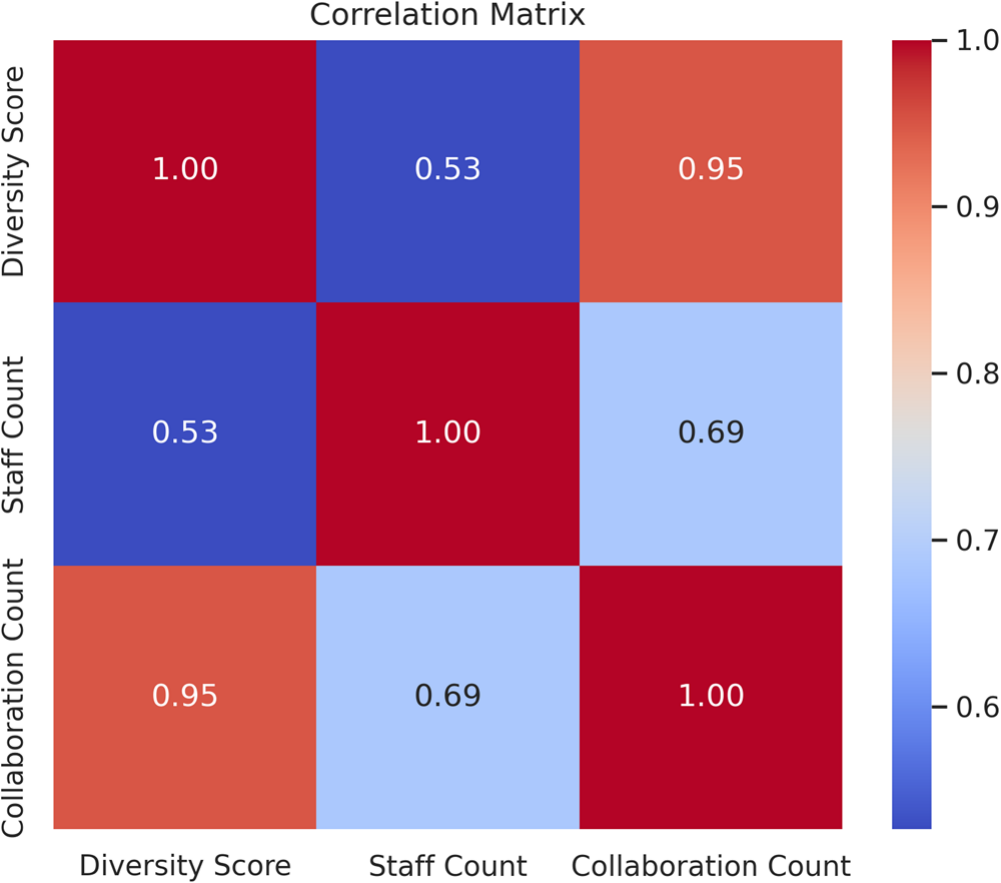
Correlation matrix of key variables related to diversity and collaboration.

**Figure 3:**
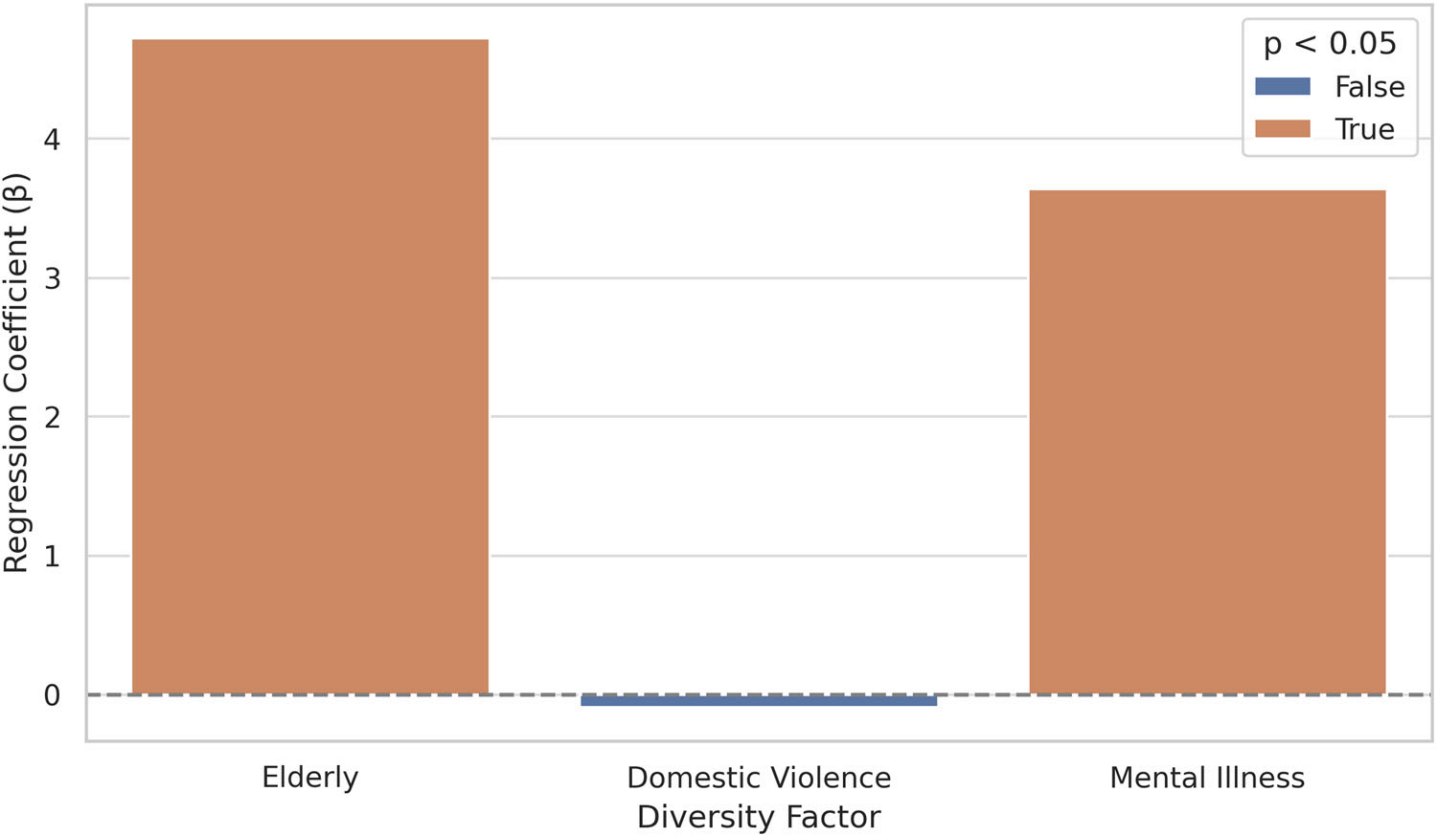
Standardized regression coefficients: effects of diversity factors on collaboration (*N* = 898).

Figure S1 shows that the strongest co‐occurrence connections were observed between Child Guidance Centers and Mental Health and Welfare Centers. In contrast, institutions such as Developmental Disabilities Centers had more limited ties. The full co‐occurrence matrix is shown in Table S1. Cluster 1 institutions reported greater collaboration with medical and welfare services, whereas Cluster 0 institutions had limited ties beyond internal organizational resources. Figures 1, 2 and 2 summarize distributions and associations among key variables; cluster‐level contrasts are detailed in Table S1 (Figure 3).

### Additional findings from network and cluster‐based visualization

**Figure 4:**
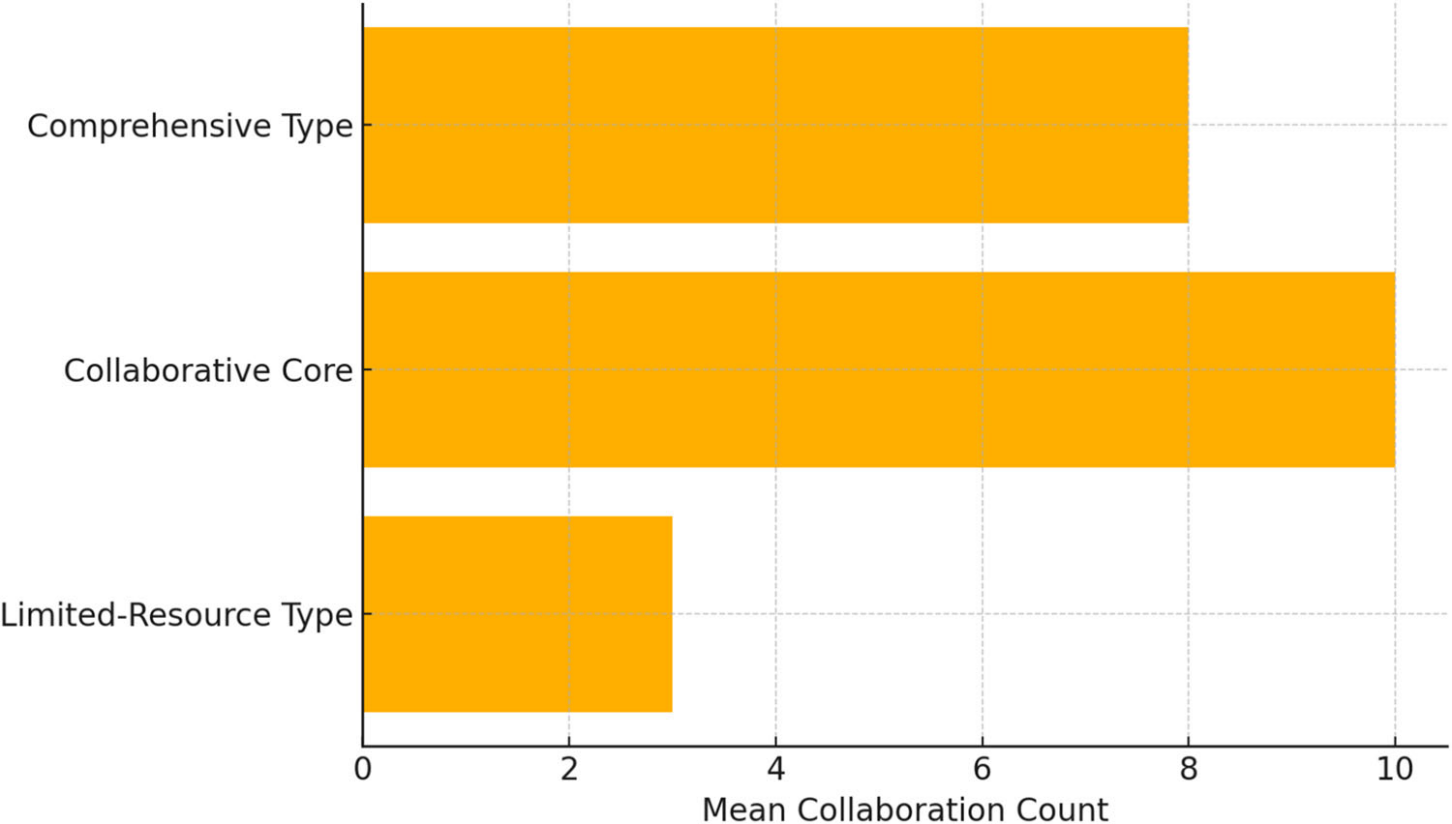
Average number of external collaborations by facility type. Because some facilities have multiple functions, category totals may exceed the overall sample size (*N* = 898).

**Figure 5:**
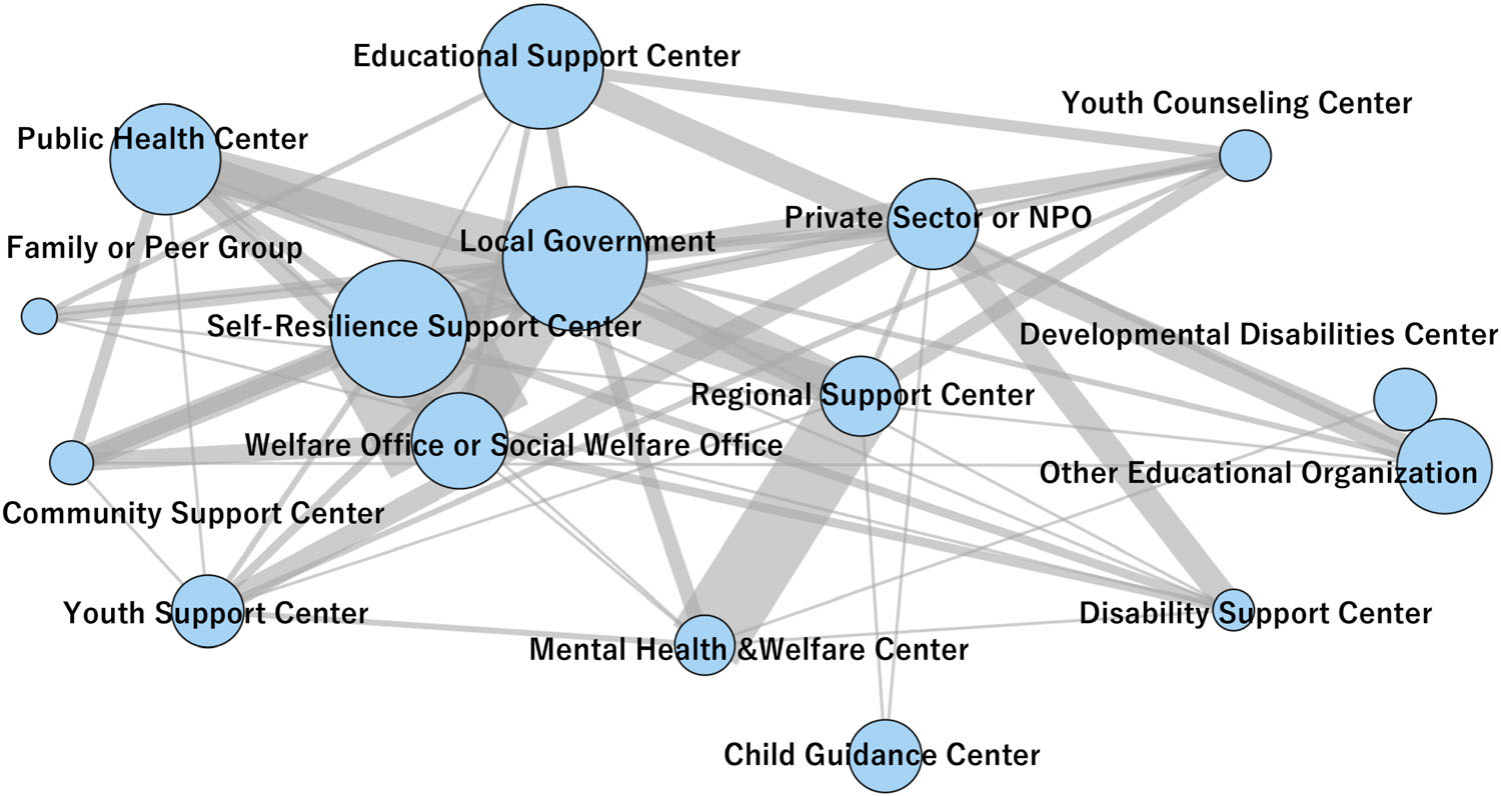
Network diagram of facility collaboration across 16 partner types.

Figure 5 presents the overall inter‐institutional collaboration network. As shown in Figure 4, child guidance and mental health and welfare centers reported the highest average number of collaborations. The variation in partner types across clusters is visualized in Figure 5, which illustrates the frequencies of collaboration across clusters and partner types.

## DISCUSSION

The three identified clusters are termed the limited‐resource type (Cluster 0), collaborative core (Cluster 1), and comprehensive type (Cluster 2). These labels reflect the differences in resource availability, collaboration style, and operational scale. Collaborative Core facilities are characterized by moderate staff and case volumes but demonstrate high levels of interorganizational collaboration, often acting as agile coordinators. In contrast, large‐scale, comprehensive facilities provide broad services with substantial human resources and caseloads. While Cluster 1 institutions demonstrate structural centrality and wider service provision, this centrality is supported by their higher median number of collaborations and coverage of consultation and outreach functions (see Figure 2).^35^

These structural insights imply that Cluster 0 facilities, which often lack medical affiliations and external partnerships, may benefit from targeted funding and human resource support. These structural differences across clusters are illustrated in Figures 1 and 2 and summarized in Table S1.

### Temporal context and generalizability

This study analyzed institutional data collected in early 2021, during the COVID‐19 pandemic. While the pandemic may have temporarily affected service delivery patterns, the structural characteristics we examined—staffing composition, target population diversity, and interorganizational networks—represent relatively stable organizational features that are less susceptible to short‐term disruptions. The policy framework we propose focuses on these durable structural attributes rather than transient operational metrics.

### Staff size and institutional function

This finding suggests that for hikikomori support, simply increasing personnel numbers may not automatically lead to better interagency networking. Instead, functional characteristics—specifically the capability to handle diverse cases (Target Diversity) and the accumulation of practical experience (Case Volume)—appear to be more critical drivers of collaboration than organizational size. This underscores the importance of “functional hubs” capable of addressing complex needs, rather than just “large” institutions.

### How target diversity shapes collaboration

Facilities that support a broader range of client types, such as individuals with psychiatric symptoms, developmental disorders, survivors of domestic violence, or older adults, have shown increased collaboration with external entities. This suggests that complex or multilayered clients require multi‐sectoral engagement. These results are consistent with those of previous qualitative studies emphasizing that service fragmentation can hinder access for individuals with overlapping vulnerabilities.

### Service demand and network formation

Facilities that manage more cases tend to be more collaborative. While this may reflect higher operational capacity or institutional centrality, it may also indicate reactive strategies for managing increased service demand by extending networks. However, as staff size is highly correlated with case volume, further research is needed to assess whether increased collaboration arises from proactive strategic positioning or is merely a byproduct of case overload.

### Typology of facility operations

Our cluster analysis yielded three distinct facility profiles: (1) limited‐resource, low‐collaboration facilities; (2) moderately resourced facilities with high collaboration; and (3) large‐scale, high‐capacity institutions. These typologies reflect variations in size, scope, and strategic orientations. Despite having limited staff, facilities in Cluster 1 are remarkably active in collaboration, suggesting that efficient networking and outreach may compensate for limited internal resources.

This typology provides a potential framework for policy design, allowing for tailored interventions depending on a facility's resource level and collaboration style. For instance, the facilities in Cluster 0 may benefit from targeted investments in coordination roles, whereas Cluster 1 facilities could be supported through capacity building to manage growing caseloads without compromising quality.

### Network‐based interpretation

Our visualization of the institutional collaboration network further supports the structural centrality of Cluster 1 facilities. These lean, outreach‐oriented institutions are positioned as dense nodes within the network, highlighting their potential roles as regional brokers and integrators of fragmented services.

Importantly, our findings suggest that institutional size and specialization alone do not account for collaborative engagement. Instead, network density and positioning—what might be termed “collaborative centrality”—serve as stronger predictors of integrative capacity. The active engagement of Cluster 1 institutions with non‐traditional partners, such as NPOs and youth employment centers, points to their flexibility and responsiveness to complex cases of social withdrawal. These insights underscore the importance of investing in structural connectivity and intersectoral openness in future system‐level planning.

### Public health implications

The coexistence of typological diversity and relational network structures indicates a dual pathway for capacity development in public mental health systems. Developed in the Japanese context, this framework may be scalable to other countries dealing with similar youth withdrawal issues, particularly in East Asian and middle‐income settings. Facilities with strong cross‐sectoral collaborations, especially those acting as regional hubs, should be prioritized for strategic investments. Lean institutions with agile operations and outreach capacities, identified as Cluster 1, emerged as key coordination hubs. Policymakers should consider investing in such institutions to strengthen regional collaboration and reduce fragmentation in service provision. Our findings suggest that collaboration density and network centrality, rather than institutional size or specialization, better predict effective service coordination. Encouraging diverse client engagement and cross‐sector partnerships (including non‐traditional actors such as NPOs and youth employment support centers) may help extend the reach of hikikomori services. This typological and relational approach offers a replicable framework for policy design in other decentralized health systems facing similar challenges in serving marginalized populations. It also supports global public health goals of improving inclusion, accessibility, and system resilience.

### Strengths and limitations

This study had several limitations. First, the data were collected through self‐administered questionnaires, which may have been subject to response bias. Second, the cross‐sectional design does not allow for causal inferences between diversity, collaboration, and institutional characteristics. Third, this analysis may not fully account for regional disparities or urban–rural differences. Fourth, the definition and scope of hikikomori support may vary across facilities, leading to inconsistencies in reporting. Finally, the diversity and collaboration indices used in this study were developed specifically for this research and warrant further validation in future studies.

As responses were self‐reported, non‐differential misclassification is likely and would bias estimates toward the null, which is consistent with the modest *R* ^2^. Reporting cultures may vary across sectors; triangulation with administrative records and referral logs in future studies would mitigate this limitation.

### Implications for policy and practice

Evidence from this national sample suggests that investing in lean, outreach‐oriented institutions as regional hubs may reduce fragmentation and improve equity of access. Strengthening intersectoral governance across health, welfare, education, and employment aligns with universal health coverage and Sustainable Development Goal (SDG) 3/10. Our typology offers a practical framework for workforce planning and network design that can be adapted to other decentralized settings. Future implementation studies should report costs, reach, and quality‐of‐care outcomes to assess scalability.

## CONCLUSIONS

This nationwide study highlights that client diversity and service volume are the primary structural drivers of external collaboration, outweighing the influence of staff size. The identification of three distinct institutional clusters reveals the substantial structural heterogeneity within Japan's hikikomori support system. Specifically, lean, outreach‐oriented facilities (Cluster 1) serve as critical regional coordination hubs. These findings provide an evidence‐based typology to inform resource allocation and reduce service fragmentation for socially withdrawn populations.

## AUTHOR CONTRIBUTIONS

**Masahide Usami**: Conceptualization; methodology, formal analysis, writing—original draft. **Yuki Mizumoto**: Data curation; investigation. **Kumi Inazaki**: Writing—review and editing; supervision. **Yuki Hakoshima**: Writing—review and editing; supervision. **Kotoe Itagaki**: Writing—review and editing; supervision. **Keita Yamamoto**: Writing—review and editing; supervision. **Tetsushi Tsujimoto**: Writing—review and editing; supervision. **Masao Yamasaki**: Writing—review and editing; supervision. **Kazuhiko Saito**: Writing—review and editing; supervision. All authors reviewed and approved the final manuscript.

## CONFLICT OF INTEREST STATEMENT

The authors declare no conflicts of interest.

## ETHICS APPROVAL STATEMENT

Not applicable. This study used only non‐identifiable, institutional‐level data and did not involve human participants, human data, or human tissue.

## PATIENT CONSENT STATEMENT

N/A.

## CLINICAL TRIAL REGISTRATION

N/A.

## Supporting information

Supporting Information.

## Data Availability

Data sharing is not applicable to this article as no datasets were generated or analyzed during the current study. De‐identified survey data are available from the corresponding author on reasonable request.
