# Supplementary material for: Identifying hub functions and collaboration patterns in hikikomori support networks: A nationwide cross‐sectional study
Source: PCN Rep. 2026 Mar 1;5(1):e70311. doi: 10.1002/pcn5.70311 (PMC12949849; doi:10.1002/pcn5.70311)
Supplement: Supplementary file 1 — Supporting Information. [file PCN5-5-e70311-s001.docx]

## Additional Files

Additional file 1: Table S1. Summary Statistics of Institutional Clusters: Staff Composition, External Collaboration, and Hikikomori Case Volume.

Additional file 2: Table S2. Sensitivity analysis (N = 902): standardized coefficients, 95% confidence intervals, and p-values.

| **Term** | **Std. Coef (β)** | **95% CI** | **p-value** |
| --- | --- | --- | --- |
| Target diversity | 0.111 | [0.048, 0.173] | 5.4e-04 |
| Case volume (FY2019, total) | 0.141 | [0.076, 0.206] | 2.4e-05 |
| Total staff (hikikomori team) | -0.001 | [-0.065, 0.064] | 0.981 |

Additional file 3: Figure S1. Co-occurrence Network of Collaboration Among 16 Facility Types.

Additional file 4: Figure S2. Heatmap of collaboration frequency by cluster and partner type. Cluster sizes: C0 = 252, C1 = 301, C2 = 345 (total N = 898). Note: Because some facilities have multiple functions, category totals may exceed the overall sample size (N = 898).

Additional file 5: Figure 5. Network Diagram of Facility Collaboration Across 16 Partner Types.
